# Synergistic Effect of WTC-Particulate Matter and Lysophosphatidic Acid Exposure and the Role of RAGE: In-Vitro and Translational Assessment

**DOI:** 10.3390/ijerph17124318

**Published:** 2020-06-17

**Authors:** Rachel Lam, Syed H. Haider, George Crowley, Erin J. Caraher, Dean F. Ostrofsky, Angela Talusan, Sophia Kwon, David J. Prezant, Yuyan Wang, Mengling Liu, Anna Nolan

**Affiliations:** 1 Division of Pulmonary, Department of Medicine, Critical Care and Sleep Medicine, New York University (NYU) School of Medicine, New York, NY 10016, USA; rachel.lam@nyulangone.org (R.L.); SyedHissam.Haider@nyulangone.org (S.H.H.); george.crowley@nyulangone.org (G.C.); Erin.Caraher@nyulangone.org (E.J.C.); Dean.Ostrofsky@nyulangone.org (D.F.O.); talusa01@nyu.edu (A.T.); sophia.kwon@nyulangone.org (S.K.); 2 Bureau of Health Services and Office of Medical Affairs, Fire Department of New York, Brooklyn, NY 11201, USA; david.prezant@fdny.nyc.gov; 3 Pulmonary Medicine Division, Department of Medicine, Montefiore Medical Center and Albert Einstein College of Medicine, Bronx, NY 10461, USA; 4 Division of Biostatistics, Departments of Population Health, New York University School of Medicine, New York, NY 10016, USA; Yuyan.wang@nyulangone.org (Y.W.); mengling.liu@nyulangone.org (M.L.); 5 Department of Environmental Medicine, New York University School of Medicine, New York, NY 10016, USA

**Keywords:** lysophosphatidic acid, particulate matter exposure, RAGE, synergy

## Abstract

World Trade Center particulate matter (WTC-PM)-exposed firefighters with metabolic syndrome (MetSyn) have a higher risk of WTC lung injury (WTC-LI). Since macrophages are crucial innate pulmonary mediators, we investigated WTC-PM/lysophosphatidic acid (LPA) co-exposure in macrophages. LPA, a low-density lipoprotein metabolite, is a ligand of the advanced glycation end-products receptor (AGER or RAGE). LPA and RAGE are biomarkers of WTC-LI. Human and murine macrophages were exposed to WTC-PM, and/or LPA, and compared to controls. Supernatants were assessed for cytokines/chemokines; cell lysate immunoblots were assessed for signaling intermediates after 24 h. To explore the translatability of our in-vitro findings, we assessed serum cytokines/chemokines and metabolites of symptomatic, never-smoking WTC-exposed firefighters. Agglomerative hierarchical clustering identified phenotypes of WTC-PM-induced inflammation. WTC-PM induced GM-CSF, IL-8, IL-10, and MCP-1 in THP-1-derived macrophages and induced IL-1α, IL-10, TNF-α, and NF-κB in RAW264.7 murine macrophage-like cells. Co-exposure induced synergistic elaboration of IL-10 and MCP-1 in THP-1-derived macrophages. Similarly, co-exposure synergistically induced IL-10 in murine macrophages. Synergistic effects were seen in the context of a downregulation of NF-κB, *p*-Akt, -STAT3, and -STAT5b. RAGE expression after co-exposure increased in murine macrophages compared to controls. In our integrated analysis, the human cytokine/chemokine biomarker profile of WTC-LI was associated with discriminatory metabolites (fatty acids, sphingolipids, and amino acids). LPA synergistically elaborated WTC-PM’s inflammatory effects in vitro and was partly RAGE-mediated. Further research will focus on the intersection of MetSyn/PM exposure.

## 1. Introduction

Particulate matter (PM) exposure has been increasingly linked to systemic disease and lung pathology [1,2,3,4]. Furthermore, PM exposure significantly increases low-density lipoprotein (LDL), cholesterol, and triglycerides, and reduces high-density lipoprotein (HDL) levels [5,6]. Cross-sectional studies have found strong associations between PM exposure, loss of lung function, and metabolic syndrome (MetSyn), a cluster of risk factors for cardiovascular diseases such as hypertension, dyslipidemia, and insulin resistance [7]. We have previously identified that dyslipidemia and insulin resistance in World Trade Center (WTC)-PM-exposed Fire Department of New York (FDNY) first responders increased the risk of developing WTC Lung Injury (WTC-LI) [8,9]. Our murine and FDNY WTC-PM-exposed cohort studies suggest that the receptor for advanced glycation end-products (RAGE; also known as the advanced glycation end-product receptor (AGER) when referring to the human or murine protein) and lysophosphatidic acid (LPA) have key roles in the development of WTC-LI [9,10,11,12,13,14,15,16,17].

RAGE is a multi-ligand receptor of lipid and glucose metabolism intermediates, such as advanced glycation end-products (AGEs) and LPA, and contributes to inflammation, vascular injury, and pulmonary disease [18,19,20,21,22,23,24,25]. Stimulation of RAGE results in the activation of some proteins, such as NF-κB, STATs and Akt [25,26]. RAGE is expressed at low baseline levels in most other end-organs except the lung, where it is most highly expressed. It was also found to have increased expression in explanted lungs of COPD subjects [27]. Single-nucleotide polymorphisms within the RAGE gene (called AGER in these reports) were associated with FEV_1_/FVC ratio (*p* < 10^−15^) in two genome-wide association studies of 74,564 and 20,820 individuals [20,28]. 

As a member of the immunoglobulin super family, RAGE exists both in a cell-membrane-bound and a soluble form. Airway inflammation in asthma and COPD is associated with reduced levels of circulating soluble RAGE or sRAGE [29,30]. Thus, RAGE is an attractive target for potential therapeutic agents in treatment for obstructive airway disease, especially since current treatments that minimize disease progression are limited. A finer understanding of the underlying inflammation and a focus on new therapeutic targets such as the LPA/RAGE axis is crucial. 

WTC-PM exposure, dyslipidemia, and elevated LPA have been associated with the development of WTC-LI [15,16,31]. LPA is a phospholipid and is soluble in both cell membranes and in aqueous fluid that can activate pathways involved in vascular injury [32,33,34,35]. The production of LPA results from various pathways, but most involve the catabolism and oxidation of low-density lipoprotein (LDL) [36,37]. Autotaxin (Atx)/lysoPLD is a secreted enzyme that is responsible for the vast majority of LPA synthesis [38,39]. Tissue LPA concentrations are tightly regulated via modulation of Atx. [40,41]. Circulating LPA is rapidly turned over by lipid phosphate phosphatases (LPPs), which terminate its signal by dephosphorylation [42]. Pulmonary vascular injury occurs early in COPD with pulmonary perfusion abnormalities and reduced blood return to the heart observed prior to development of abnormal FEV_1_ [43,44]. Pulmonary arteriopathy was present in 58% of lung biopsies from non-FDNY WTC-PM-exposed individuals and in 74% with constrictive bronchiolitis after inhalational exposures during military service [45,46]. 

G-protein-coupled receptors (GPCRs) have been identified as specific for LPA (LPA_1–5_) [47]. Although most reported cellular responses to LPA have been attributed to cell surface GPCR activation, not all LPA activities can be explained by GPCR signaling [48,49,50]. LPA can also bind the nuclear peroxisome proliferator-activated receptor gamma (PPARγ) and initiate early stages of atherosclerosis [51]. LPA is an agonist of PPARγ [51]. Exogenous LPA might also enter cells to activate PPARγ. Specifically, when LPA enters RAW264.7 cells, it activates a reporter driven by a PPARγ expression vector [50]. 

Alveolar macrophages comprise approximately 90% of alveolar immune cells and can release inflammatory cytokines/chemokines when exposed to PM. Alveolar macrophages express RAGE, making them potentially relevant in the MetSyn/lung injury pathways [52,53]. In our earlier work, we utilized an in-vitro model of macrophages and showed that WTC-PM exposure produced comparable inflammatory profiles to those of firefighters with WTC-LI [9,54].

We now focus on the intersection of WTC-PM/lipid co-exposure [55,56]. Using a multiomic approach and murine and human in-vitro exposures, we identified key cytokines/chemokines and transcription factor profiles of WTC-PM exposure. In addition, we determined which biomarkers were synergistically induced by PM/LPA co-exposure in both human and murine macrophages. To further our translational understanding, we then integrated identified biomarkers from our in-vitro analyses with our metabolomics analysis of the PM-exposed firefighters. The incorporation of in-vitro models allows us to further understand the translatability of PM exposure responses and of pathways that may be key to loss of lung function in vivo due to PM exposure.

## 2. Methods

### 2.1. Cell Lines

To provide a macrophage phenotype, human THP-1-derived macrophage (ATCC) cells were differentiated with 20 ng/mL PMA (Sigma-Aldrich^®^, St. Louis, MO, USA) for 72 h prior to exposure. THP-1-derived macrophages were phorbol-12-myristate-13-acetate (PMA)-differentiated and cultured in RPMI1640 (Gibco) and murine RAW264.7 (ATCC) (murine) macrophage-like cells were cultured in DMEM (ATCC). All cell lines were supplemented with 2 mM l-glutamine, 10% fetal calf serum (FCS) (Sigma Aldrich^®^), 100 U/mL penicillin, and 100 µg/mL streptomycin (Cellgro) at 37 °C, 5%CO_2_. Trypan blue (Thermo Scientific^TM^, MA, USA) exclusion assessed cell viability [57]. 

### 2.2. WTC-PM and LPA Preparation

WTC-PM_53_ (≤53 µm) was collected in bulk, aerosolized, sieved using a 53-µm diameter mesh, analyzed as previously published, and stored at room temperature as previously described [15,58]. LPA (lysophosphatidic acid 18:1; CAS 65528-98-5) was purchased from Santa Cruz^®^ (Dallas, TX, USA) (sc-222720) and supplied as a solution in ethanol. After ethanol evaporation, LPA was reconstituted in media to a final concentration of 500 µM per well. The 500 µM LPA dosing was chosen based on preliminary dose-finding experiments that showed a synergistic elaboration that was not present at lower doses (data not shown). This dose also has clinical plausibility, as we have previously evaluated firefighters with WTC exposure and found that their LPA levels exceed 200 µM, higher than previously recorded levels of healthy patients [31,59]. This dose has also been found to inhibit humoral immunity signaling [60].

### 2.3. Exposures

THP-1-derived macrophages and RAW264.7 cells were exposed to WTC-PM_53_ suspensions (50 μg/mL or 100 μg/mL) and/or 500 µM of LPA. All subsequent experiments utilized only the 100 μg/mL dose of WTC-PM since prior work showed it could induce a profound inflammatory response and closely emulate real-world delivery of WTC-PM [9,15,61]. Macrophages were plated at 1 × 10^6^ cells/mL in 12-well plates, 1.5mL total volume. Adherent macrophages were exposed to PBS, 100 µg/mL suspensions of WTC-PM_53_, 500 µM of LPA, (Santa Cruz^®^) or both WTC-PM_53_ and LPA. After 24 h, supernatants collected and stored at −80 °C [62,63]. All experiments done in triplicate and controlled for volume.

### 2.4. Immunoblots

Cultured cells (THP-1-derived macrophages and RAW264.7) were lysed in NP-40 lysis buffer. This is composed of 1% NP-40 (Sigma), 20% Glycerol (Santa Cruz^®^), 0.2 mM EDTA (Sigma), 40 mM HEPES (Gibco) pH 7.9, 0.5 M NaF (Sigma), 10 mM NaPpi (Sigma), 5 M NaCl (Fisher) and was supplemented with protease inhibitor cocktail (aprotinin, leupeptin, and pepstatin in a 1:1:1 ratio; Sigma), 0.5 M dithiothreitol (Sigma), 10 mg/mL phenylmethanesulfonyl fluoride (Sigma) and 100 mM Na_3_VO_4_ (Sigma). Debris was removed by centrifugation (Sorvall) at 1500 rpm for 15 min, and proteins were fractionated by SDS-PAGE, 8% polyacrylamide gel (BIO-RAD), and probed for RAGE, JNK, *p*-Akt, *p*-STAT3, STAT5b, and GAPDH (Santa Cruz^®^). These were visualized with horseradish-peroxidase-coupled 2° antibodies—mouse-IgGκ, goat anti-rabbit IgG, or donkey anti-goat IgG (Santa Cruz^®^) [62,64,65]—developed using western blotting substrates (Thermo Scientific^TM^ Pierce^TM^ ECL Plus Western Blotting Substrate, Rockford, IL, USA), and image-captured (FluorChem^TM^ 8900, ProteinSimple, San Jose, CA, USA). All experiments were done in triplicates. Densitometric analyses (ImageJ, version 1.53a [66]) were performed. Each band was quantified five times, and we reported the fold change of each exposure condition compared to media alone (MA), as described [63].

### 2.5. Cytokine/Chemokine Assessment

Supernatants from THP-1-derived macrophages were assayed using a human cytokine/chemokine multiplex panel (Millipore; #HCYTMAG-60K-PX29. Analytes: EGF; G-CSF; GM-CSF; IFN-α2; IFN-γ; IL-1α; IL-1β; IL-1ra; IL-2; IL-3; IL-4; IL-5; IL-6; IL-7; IL-8; IL-10; IL-12 (p40); IL-12 (p70); IL-13; IL-15; IL-17A; IP-10; MCP-1; MIP-1α; MIP-1β; TNF-α; TNF-β; VEGF; Eotaxin). RAW264.7 cell line supernatants were assayed on a murine cytokine/chemokine panel (Millipore; #MCYTOMAG-70K-PX25. Analytes: G-CSF; GM-CSF; IFN-γ; IL-1α; IL-1β; IL-2; IL-4; IL-5; IL-6; IL-7; IL-9; IL-10; IL-12 (p40); IL-12 (p70); IL-13; IL-15; IL-17; IP-10; KC; MCP-1; MIP-1α; MIP-1β; MIP-2; RANTES; TNF-α). Both were assayed on a 200IS (Luminex) and analyzed (MasterPlex-QT; MiraiBio). Analytes that fell within the manufacturer’s recommended range of detection were presented in the results.

### 2.6. NF-κB Assay

RAW264.7 cell lysates’ NF-κB (p65) were quantified using DNA-binding ELISA (Cayman Chemical MI, USA; Item No. 100007889). Plate and buffers were brought to room temperature. All steps outlined by the manufacturer were followed and included the use of recommended controls. Absorbances were read at 450 nm (FluoSTAR Optima; BMJ Labtech). Fold change of exposure compared to PBS control was calculated for all. 

### 2.7. Study Design, Serum Analytes and Metabolomics

Cases of WTC-LI (*n* = 15) and controls (*n* = 15) were drawn from a previously described cohort of firefighters referred for subspecialty pulmonary examination [9,11,15,67,68,69]. Briefly, non-smoking firefighters with normal pre-9/11 lung function were followed for 16 years post-exposure to WTC-PM and were identified as having WTC-LI if FEV_1,%Predicted_ < lower limit of normal (LLN) as defined by National Health and Nutrition Examination Survey III, and controls if FEV_1,%Predicted_ ≥ LLN. Demographic and clinical data were obtained from the WTC Health Program (WTC-HP) [70,71,72,73]. All serum was collected within 200 days of 11 September 2001 and was aliquoted and stored at −80 °C [9,10,67,68,73]. Analytes and the metabolome were assessed as previously described [73,74,75]. 

### 2.8. Integration

A subset of maximally discriminative metabolites previously identified via a machine learning algorithm, random forests, were integrated with the human analogues of significantly altered cytokines/chemokines identified from this manuscript’s in vitro studies [15,31,67,73,74]. Agglomerative hierarchical clustering was performed on standardized data with Spearman’s rank correlation matrices and average linkage (Matlab, Mathworks).

### 2.9. Statistical Analysis

SPSS-23 (IBM, Armonk, NY, USA) and *Prism* 5.0 (GraphPad, San Diego, CA, USA) were used for data storage, handling and analysis. Cytokine/chemokine concentrations were expressed as medians (IQR) and compared by the Mann–Whitney U-test. For categorical data, counts and proportions were used, while the Pearson-χ^2^ test was applied for comparison. The interaction (additive and synergistic) between WTC-PM_53_ and LPA was assessed by two-way ANOVA, corrected for multiple comparisons by Sidak’s test [57,76,77]. Results were deemed statistically significant at *p* < 0.05. 

### 2.10. Ethics Approval and Consent to Participate

We have reviewed the journal’s guidelines involving ethical publication and we confirm here that we have abided by said guidelines. All subjects, at the time of enrolment, consented to analysis of their information and samples for research according to Institutional Review Board approved protocols at Montefiore Medical Center (#07-09-320) and New York University (#16-01412).

## 3. Results

### 3.1. WTC-PM and LPA Exposure Induce Analyte Elaboration in THP-1-Derived Macrophages

**Figure 1:**
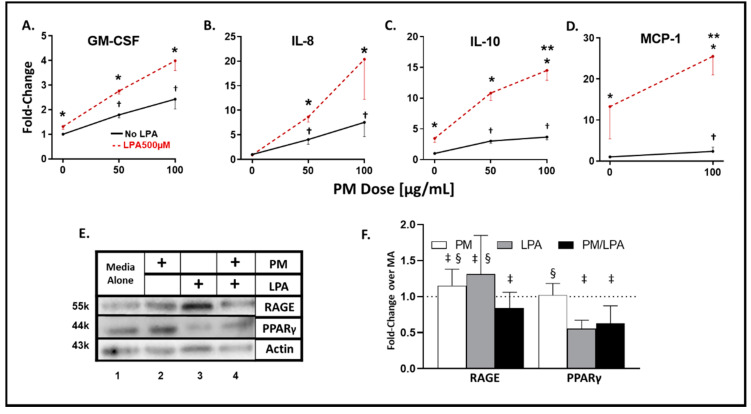
Analyte and transcription factor response to WTC-PM and LPA in THP-1-derived macrophages. Differentiated THP-1 cells were used for all panels. Supernatants were assayed after 24 h of WTC-PM and/or LPA exposure. (**A**) GM-CSF (**B**) IL-8 (**C**) IL-10 (**D**) MCP-1. All values reported as mean ± SD of fold change over PBS. (**A**–**C**) 0, 50 and 100 μg/mL WTC-PM/LPA co-exposure-induced dose-dependent cytokine/chemokine elaboration response in cell supernatants, *n* = 3. (**A**–**D**) WTC-PM-induced synergistic elaboration of (**C**) IL-10 in THP-1 cell culture supernatant after WTC-PM/LPA co-exposure. Independent LPA exposure is denoted as the left red point. (**E**) Immunoblots display RAGE, PPARγ, and actin expression: Lane 1 Media Alone, Lane 2 WTC-PM, Lane 3 LPA and Lane 4 WTC-PM/LPA. (**F**) Densitometry analyses of immunoblots: fold change over media alone (MA). * denotes *p* < 0.05 between LPA and no LPA, Student’s *t*-test; ** *p* < 0.05 for interaction of WTC-PM/LPA co-exposure, multiple *t*-tests by row; † *p* < 0.05 compared to PBS, Student’s *t*-test; ‡ *p* < 0.05 compared to media alone, Student’s *t*-test; § *p* < 0.05 compared to WTC-PM/LPA co-exposure, Student’s *t*-test.

In THP-1-derived macrophages, WTC-PM exposure induced significant, dose-dependent (50μg/mL fold change (*p*-value) vs. 100 μg/mL fold change (*p*-value)) increases in GM-CSF (1.79(0.01) vs. 2.43(0.002)), IL-8 (1.79(0.002) vs. 2.43(0.002)), and IL-10 (3.02(0.01) vs. 3.67(0.002)) relative to PBS levels (Figure 1A–C). LPA exposure induced significant (fold-change (*p*-value)] elaboration of GM-CSF (1.32(0.01)) and IL-10 (3.45(0.01)) relative to PBS (Figure 1A–C and Table 1). When comparing WTC-PM exposure to PBS, there was a significant increase in G-CSF, GM-CSF, IFN-γ, IFNA2, IL-1β, IL-7, IL-8, IL-10, IL-15, TNF-α, IL-12(p40, p70), Eotaxin, IP-10, MCP-1, MIP-1α, and MIP-1β (Table 1). When comparing LPA exposure to PBS, there was a significant increase in G-CSF, GM-CSF, IL-7, IL-8, IL-10, IL-15, VEGF, IL-12(p40, p70), Eotaxin, MCP-1, and MIP-1α. In contrast, there was inhibition of IP-10 (Table 1).

### 3.2. Synergistic Response to WTC-PM/LPA Co-Exposure in Human-THP-1-Derived Macrophages

A dose-dependent elaboration response was observed in the WTC-PM/LPA co-exposure cohort for GM-CSF, IL-8, and IL-10 (Figure 1A–C). When comparing independent WTC-PM to co-exposure of WTC-PM/LPA, there was a significant increase in GM-CSF, IL-8, VEGF, MCP-1 and MIP-1α. However, there were also inhibitory effects seen in Eotaxin, IP-10, and MIP-1β (Table 1).

WTC-PM/LPA co-exposure displayed a significant synergistic increase in IL-10 (14.50(< 0.0001)) and MCP-1 (24.93(0.0081)) elaboration relative to independent WTC-PM or LPA exposure (Figure 1C,D). Multiplex data are provided in Table 1.

Analytes IL-1α, IL-2, IL-3, IL-4, IL-5, IL-6, IL-13, IL-17A, and TNF-β were below the manufacturer’s lower limit of detection and therefore not shown. 

### 3.3. WTC-PM and LPA Induce RAGE/PPARγ Protein Production in Human-THP-1-Derived Macrophages

Levels of RAGE and PPARγ were assessed to determine the direct cellular effects. Compared to MA, WTC-PM independently induced RAGE and PPARγ protein production in differentiated THP-1-derived macrophages (Figure 1E, Lanes 1,2). LPA independently induced RAGE (Figure 1E, Lanes 1,3). WTC-PM/LPA co-exposure reduced RAGE and PPAR-γ production relative to independent exposures (Figure 1E, Lanes 1–4). WTC-PM and LPA independently induced significantly higher RAGE protein production compared to MA, whereas co-exposure decreased RAGE by densitometry (Figure 1F). Moreover, WTC-PM and LPA each had significantly higher RAGE production compared to co-exposure. PPAR-γ did not change after WTC-PM exposure, whereas it was decreased after LPA and co-exposure (Figure 1F). Similarly, co-exposure significantly reduced PPAR-γ production compared to WTC-PM alone. 

### 3.4. WTC-PM and LPA Exposures of RAW264.7 Cells Yielded Analyte Elaboration

**Figure 2:**
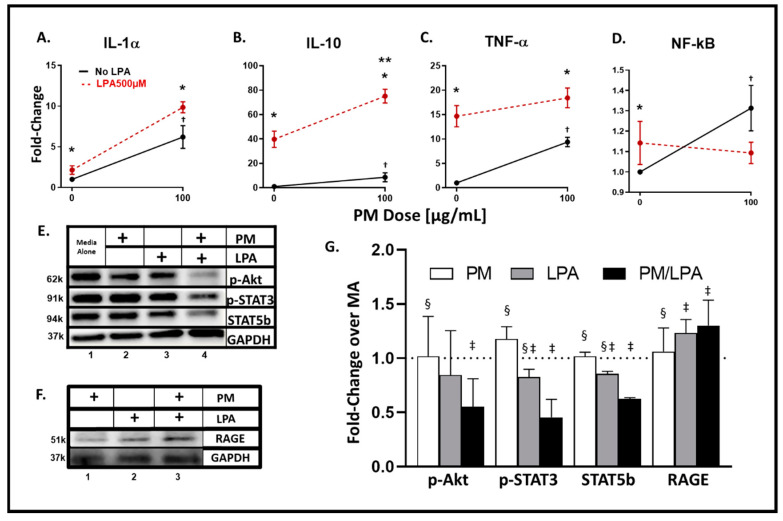
Analyte and transcription factor response to WTC-PM and LPA in RAW264.7 Supernatants were assayed after 24 h of WTC-PM and/or LPA exposure, *n* = 3. (**A**) IL-1α, (**B**) IL-10, (**C**) TNF-α, (**D**) NF-κB. All values reported as mean ± SD of fold change over PBS. Independent LPA exposure is denoted as the left red point. (**A**–**D**) Synergistic inflammatory expression of (**B**) IL-10 observed after WTC-PM/LPA co-exposure. (**D**) PM-induced NF-κB elaboration greater than that of PM/LPA co-exposure. (**E**) Immunoblots display *p*-Akt, *p*-STAT3, STAT5b, and GAPDH expression: Lane 1 Media Alone, Lane 2 WTC-PM, Lane 3 LPA, Lane 4 WTC-PM/LPA. (**F**) Immunoblots display RAGE and GAPDH expression: Lane 1 WTC-PM, Lane 2 LPA, Lane 3 WTC-PM/LPA. (**G**) Densitometry analyses of immunoblots; fold change over media alone (MA). * denotes *p* < 0.05 between LPA and no LPA, Student’s *t*-test; ** *p* < 0.05 for interaction of WTC-PM/LPA co-exposure, multiple *t*-tests by row; † *p* < 0.05 compared to PBS, Student’s *t*-test; ‡ *p* < 0.05 compared to media alone, Student’s *t*-test; § *p* < 0.05 compared to WTC-PM/LPA co-exposure, Student’s *t*-test.

Independent WTC-PM exposure at 100 μg/mL induced a significant fold change in production of RAW264.7 cell cytokines/chemokines IL-1α [6.21(0.04)], IL-10 (8.66(0.005)), and TNF-α (9.38(0.04)) relative to that of PBS exposure (Figure 2A–C). Independent LPA exposure yielded a similar significant production and analyte profile: IL-1α (4.80(0.04)), IL-10 (39.81(0.01)), and TNF-α (14.67(0.037)). RAW264.7 cells exposed to WTC-PM exhibited significant NF-κB fold change production (Figure 2D). Analyte levels and significant comparisons are available in Table 2. When comparing WTC-PM exposure to PBS, there was a significant increase in G-CSF, GM-CSF, IFN-γ, IL-1α, IL-4, IL-5, IL-6, IL-9, IL-13, IL-15, IL-17, TNF-α, IL-10, IP-10, KC, MCP-1, MIP-1α, and RANTES (Table 2). When comparing LPA exposure to PBS, there was a significant increase in IFN-γ, IL-9, IL-17, TNF-α, MCP-1, and NF-kB. In contrast, there was a significant reduction in IP-10 (Table 2). 

### 3.5. Synergistic Response to WTC-PM/LPA Co-Exposure in RAW264.7 Cells

In contrast to differentiated THP-1-derived macrophages, murine macrophages co-exposed to WTC-PM/LPA induced a synergistic expression of only IL-10 (75.11(<0.0001)) (Figure 2B). WTC-PM caused a significant increase, while co-exposure caused a decreased production of NF-κB fold change (Figure 2D). When comparing WTC-PM exposure to co-exposure of WTC-PM/LPA, there was a significant increase in IL-1α, -10, and MCP-1 (Table 2). In contrast, there was a significant reduction in the expression of IL-6, IL-17, IP-10, and RANTES (Table 2). 

Analytes IL-1β, IL-2, MIP-1α, MIP-1β and MIP-2 were below or exceeded the manufacturer’s limit of detection and therefore not shown.

### 3.6. WTC-PM and LPA Induce RAGE Protein Production in Murine RAW264.7 Cells

Immunoblot assays show a reduction in *p*-Akt, *p*-STAT3, and STAT5b expression after co-exposure (Figure 2E, Lane 4). WTC-PM alone did not modify production in any of the measured proteins when compared to MA (Figure 2G). LPA and co-exposure independently decreased *p*-STAT3 and STAT5b production when compared to MA by densitometry. Moreover, WTC-PM and LPA each had significantly higher *p*-STAT3 and STAT5b production compared to co-exposure. Additionally, co-exposure decreased *p*-Akt when compared to MA (Figure 2G). Similarly, co-exposure significantly reduced *p*-Akt production compared to WTC-PM alone. LPA induced greater RAGE expression compared to WTC-PM (Figure 2F). Moreover, unlike that found in our human macrophages, co-exposure induced RAGE production greater than that of either WTC-PM or LPA alone (Figure 2F,G). This is reflected in densitometry showing LPA and co-exposure independently inducing significantly higher RAGE production when compared to MA (Figure 2G). Additionally, co-exposure significantly increased RAGE production compared to WTC-PM alone. 

### 3.7. MultiOMIC (Metabolome and Chemome) Integrated Biomarker Analysis

Serum analyte data collected from FDNY WTC-exposed first responders for GM-CSF, IL-10, -8, -1α, MIP-1α, TNFα, LPA, and RAGE were of interest due to our in-vitro model findings in our prior work (Table 3) [9,15,31,67,68,73,78]. Serum MCP-1 exhibited a significant median difference (Table 3). These were integrated with the metabolite data assayed in the same population (*n* = 15/group).

**Figure 3:**
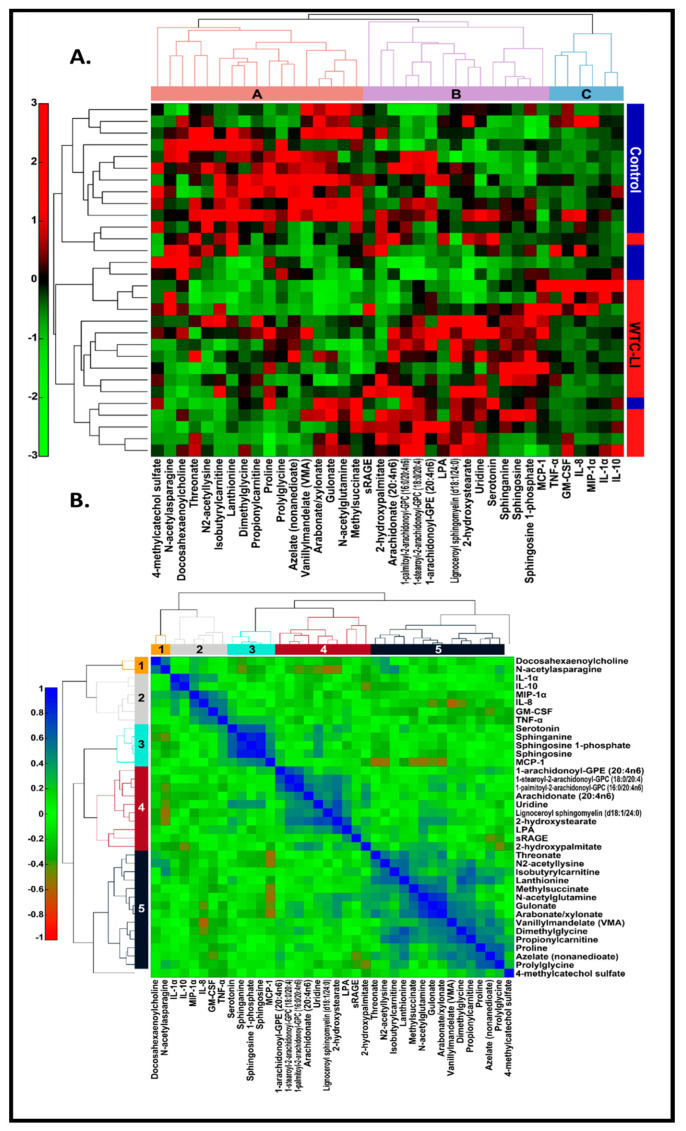
(**A**) Hierarchical clustering of 11 known WTC-LI biomarkers in *n* = 15 WTC-LI and *n* = 15 controls, Spearman’s rank correlation, and average linkage [9,15,31,67,68,73,81]. (**B**) Correlation matrix.

Linkage thresholds determined by inspection of the dendrograms were used to highlight clusters of metabolites that may reflect mechanistic relations. For the data matrix, a linkage threshold of one was used to identify three distinct clusters of metabolites (*A-C*) (Figure 3A). Cluster A consisted largely of amino acids and their metabolites, which were generally decreased in cases of WTC-LI compared to controls. These metabolites included acyl/acetylated, BCAAs, and those of the urea cycle. Meanwhile, cluster B consisted mostly of lipids and their metabolites, including the glycerol-3-phosphocholines, arachidonate, and LPA. Interestingly, in WTC-LI cases, elevated sRAGE and other metabolites were found in cluster B. Cluster C was composed of the cytokines/chemokines involved in the innate acute inflammatory response and tissue repair, and did exhibit significant differences in cases of WTC-LI compared to controls. 

A linkage threshold of 0.60 highlighted five clusters (1–5) in the correlation matrix (Figure 3B). Clusters 1–5 identified in the clustering of the correlation matrix resembled clusters A-C (Figure 3A) in composition and structure. These clusters have strong, positive inter-metabolite correlations. LPA and sRAGE clustered together in the Spearman’s rank correlation matrix (Figure 3B). While IL-1α, IL-10, MIP-1α, IL-8, GM-CSF, and TNF-α clustered together (Figure 3B, cluster 2), MCP-1 clustered with serotonin and sphingolipids (Figure 3B, cluster 3). Additionally, metabolites of several clusters bore negative correlations with other clusters. Serum biomarkers and metabolites in C1–3, including N-acetylasparagine, IL-1α, IL-10, MIP-1α, IL-8, GM-CSF, TNF-α, and MCP-1, were negatively correlated with metabolites in C4 and C5. These clusters included several acetylated amino acid metabolites—N2-acetyllysine and N-acetylglutamine—several sphingolipids and sphingomyelins, and ω fatty acids. Selected data was presented in abstract form at earlier conferences [79,80].

## 4. Discussion

The global burden of co-existing PM exposure and metabolic dysregulation is significant. Systemic inflammation caused by this co-exposure results in significant end organ compromise [82,83]. Individuals with MetSyn are predisposed to PM-exposure-associated lung injury [5,8,9,84]. These modifiable risks have proven challenging to mitigate. The interaction of MetSyn and PM exposure is a topic of considerable importance.

WTC-PM-induced GM-CSF, IL-1α, TNF-α, MCP-1, and MIP-2 further supports our prior studies suggesting that WTC-PM-exposed alveolar macrophages exhibit an inflammatory response [52,54]. This is in line with other literature showing that there were higher MCP-1 levels in COPD patients [85,86]. Both THP-1-derived macrophages and RAW264.7 cells exhibited higher LPA-associated (LPA exposure and WTC-PM co-exposure) analyte levels compared to WTC-PM and control exposures. From this study, we have demonstrated that the PM-induced inflammatory response is reproducible in macrophage lines. 

RAGE expression is higher after LPA exposure relative to WTC-PM exposure. Co-exposure decreased RAGE in THP-1-derived macrophages but it was increased in RAW264.7 macrophages. RAGE activation and expression have been demonstrated to complete a positive feedback loop upon RAGE binding, with one of its many known ligands being LPA [87,88]. To date, WTC-PM has not exhibited properties that suggest direct RAGE binding. This distinction is further supported by our observed baseline RAGE levels after WTC-PM exposure in our cell lines. While our human in-vitro model showed an increase in RAGE expression only after LPA exposure, our murine in-vitro model showed an increase in expression after PM, LPA, and joint exposure. Our previous murine models have shown that both PBS- and PM-exposed mice express RAGE, and this remains elevated even one month after exposure [15,17]. WTC-PM contains silica and heavy metals that could induce double-stranded DNA breaks and the production of reactive oxygen species, such as superoxide and peroxide [89,90,91,92,93,94,95,96,97,98,99,100]. It causes cellular stress on lung epithelium and alveolar macrophages, characterized by innate inflammation via pathways including but not limited to those not mediated by RAGE. 

We know from the literature that macrophages may exhibit an activated phenotype upon LPA exposure, and can contribute to the formation of atherosclerotic plaques and coronary artery disease [101,102,103]. LPA-activated macrophages have exhibited increased TNF-α, MCP-1, IL-8, and IL-10, among other cytokines/chemokines, and therefore appear to exhibit a mixed inflammatory/anti-inflammatory response [104]. Our results and previous findings suggest that, upon WTC-PM/LPA co-exposure, the synergistic elaboration of cytokine/chemokines with heterogeneous effects may be a consequence of both a constitutive innate response to cellular damage and subsequent macrophage activation. In addition, we know from the literature that COPD patients and those with worse airflow obstruction had higher levels of IL-10 and MCP-1 [85,86,105].

Though cytokine/chemokines characteristic of such an inflammatory response are synergistically upregulated after co-exposure, NF-κB activity does not subsequently correlate. Downregulated NF-κB, *p*-STAT3, and STAT5b, coupled with elaboration (in some cases synergistic) of inflammatory cytokines/chemokines upon WTC-PM/LPA co-exposure suggests that co-exposure may attenuate the regulatory capacity of these signaling/transcriptional components [24,25]. Future studies that evaluate inflammatory/vascular disease in the WTC-LI cohort would further clarify these relationships. 

**Figure 4:**
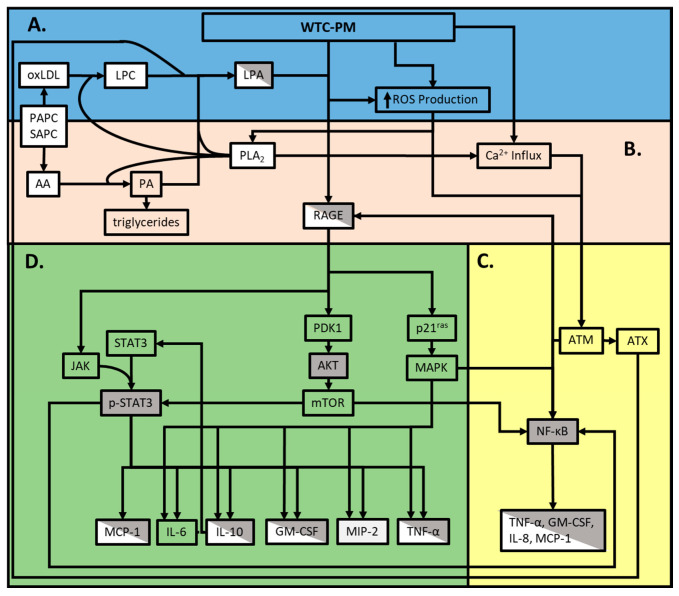
Overview of pathways involved in the WTC-PM/LPA/RAGE axis. **A.** Cytotoxic WTC-PM components induce initial cellular damage and activate innate immune responses **B.** Lipid metabolism upregulates intra- and extracellular LPA production **C.** WTC-PM exposure yields a mixed response and extracellular LPA production **D.** The LPA-RAGE axis elaborates further inflammatory and anti-inflammatory responses. Gray boxes indicate the findings of our murine assays. White boxes indicate the findings of our human assays, and diagonal shaded boxes indicates human and murine findings of this study. Clear boxes (which allow the background color to be seen) are relevant findings based on our literature review.

Establishing clusters of related biomarkers is important. Since these biomarkers may be expressed prior to phenotypic disease expression in humans, each cluster may reflect an independent pathway to disease that could be evaluated in subsequent research. High-dimensional data analysis of metabolites, serum, and clinical biomarkers support our findings, as we have identified pathways associated with the loss of lung function after WTC-PM exposure. Previous work has noted that later-staged atherosclerosis in mammalian models exhibited similar modified serum-derived amino acid metabolites. This modification of amino acid metabolites, specifically, has been associated with hypomethylation epigenetic modifications [106,107,108,109]. Elevated lipid metabolism has been linked to several pulmonary disorders and may be linked to metabolic/inflammatory lung disease and accumulation of activated macrophages. Similar to the COPD literature, we found elevated glycero-phosphatidylcholines (GPC), which are converted to LPA via cellular-damage-induced phospholipase A2 (PLA2) (Figure 4) [110,111,112,113,114,115,116,117]. We suggest that this cytokine/chemokine profile, dependent on macrophages and driven by dyslipidemia, yields subsequent WTC-LI. Based on previous literature, the amino acid cluster (5) may indicate epigenetic modification, resulting in atherosclerotic presentation, which may inhibit lung function; a correlation that could be clarified in future studies [118].

Our work has several limitations. The complexity and unique nature of both the WTC-PM and the corresponding immunological response likely incorporate multiple pathophysiological pathways, as suggested by the pathway structure (Figure 4). As per prior studies, >150 compounds have been identified in WTC-PM [119]. However, in almost all exposure-related diseases, like the sub-cohort WTC-LI, this is not the case. By utilizing WTC-PM, we limit our capacity to isolate one predominate inducer of downstream WTC-LI, but simultaneously acknowledge the complex interplay of an indefinite amount of immunologically active stimuli within the WTC-PM. Alternatively, our study focused on the co-exposure model, which encompasses elevated levels of one phospholipid derivative (LPA) observed in MetSyn as the predominate inflammatory metabolite, which works synergistically with components of WTC-PM to induce WTC-LI. In contrast to our prior studies performed in ex-vivo macrophages, MCP-1 was significantly elaborated while GM-CSF was not in our limited human sample size, Table 3 [9,54].

These multi-analyte and metabolite datasets may yield directionality in our future research associated with characterizing WTC-LI. Future studies, with respect to our approach, should focus on including more endogenous and exogenous contributors to WTC-LI and how it relates to the predisposition to this decrease in FEV_1_ in individuals suffering from MetSyn as well. Globally, air pollution contributes to pulmonary and vascular disease and complications which account for seven million annual deaths [120]. Furthermore, the prevalence of MetSyn, a vascular risk, is rapidly increasing globally, as is concurrent global high PM. The impact and resultant physiologic changes that occur in the intersection of PM exposure and MetSyn is a topic of considerable global health importance. Studies of comorbid asthma, air pollution, and MetSyn have noted that obesity-associated asthma often resists conventional therapy, further highlighting the need for novel mechanistic work [121,122,123,124]. Identifying predictive biomarkers may facilitate early treatment after PM exposure, which may be crucial to the preservation of lung function and identification of biologically plausible pathways and therapeutic targets. This work could be generalizable to biomass/PM-exposed populations since RAGE is a determinate of lung function. 

## 5. Conclusions

In conclusion, we demonstrated that WTC-PM induced inflammation in a dose-dependent manner. LPA and WTC-PM elevate RAGE protein production. Combined WTC-PM exposure and LPA synergistically enhance the observed inflammatory response. Our in-vitro findings and the WTC-LI cohort metabolomics profile suggest that the observed synergistic inflammatory response is partially a result of a dyslipidemia-driven inflammation.

## Figures and Tables

**Table 1:** Analyte Profile of THP-1-Derived Macrophages.

Analyte		PBS	WTC-PM_100μ__g/mL_	LPA	WTC-PM_100μ__g/mL_ + LPA	*p*
**Cytokine**	**G-CSF**	3.44 (3.44–4.29)	22.58 (18.71–27.21)	12.28 (12.28–15.1)	22.01 (21.72–24.79)	*, †
	**GM-CSF**	0.29 (0.25–0.41)	3.01 (2.67–3.29)	1.74 (1.43–1.83)	4.12 (3.61–4.31)	*, †, ‡
	**EGF**	6.12 (6.05–6.48)	6.89 (6.29–6.93)	6.62 (6–6.73)	7.51 (7.38–7.58)	‡
	**IFN-γ**	4.87 (4.72–5.03)	7.20 (6.81–7.20)	6.10 (5.99–6.34)	6.65 (6.65–7.01)	*
	**IFNA2**	4.74 (4.15–5.29)	18.78(17.3–20.62)	14.50 (14.06–15.37)	19.20 (17.72–21.03)	*
	**IL-1β**	9.39 (7.85–10.15)	28.39 (27.54–30.63)	11.21 (10.08–15.39)	27.84 (27.57–31.62)	*
	**IL-1RA**	403.06 (390.72–477.38)	539.47 (475.3–571.61)	455.53 (435.86–493.64)	520.76 (486.96–604.23)	
	**IL-7**	1.69 (1.62–2.07)	6.81 (6.63–7.41)	4.97 (4.16–5.71)	5.16 (4.87–6.5)	*, †
	**IL-8**	389 (334.96–396.63)	3251.24 (3039.24–3264)	1011.74 (886.89–1501.24)	3550.84 (3496.72–4043.95)	*, †, ‡
	**IL-10**	12.88 (10.38–14.53)	92.71 (67.79–157.5)	467.4 (435.4–594.8)	978.9 (861–985.7)	*, †, ‡
	**IL-15**	7.99 (7.94–7.99)	8.51 (8.41–8.51)	8.4 (8.3–8.51)	8.61 (8.51–8.66)	*, †
	**TNF-α**	11.05 (10.21–14.11)	59.75 (54.98–66.54)	16.59 (14.72–20.36)	46.2 (45.3–54.14)	*
	**VEGF**	595.13 (529.48–615.77)	674.96 (585.87–677.4)	737.59 (731.89–800.41)	828.49 (805.12–920.2)	†, ‡
	**IL-12(p40)**	6.94 (6.38–7.21)	11.62 (10.09–12.11)	9.59 (9.07–9.85)	10.61 (10.36–10.99)	*, †
	**IL-12(p70)**	5.23 (5.23–5.34)	6.00 (5.86–6.12)	5.78 (5.75–5.78)	5.78 (5.73–5.89)	*, †
**Chemokine**	**Eotaxin**	6.61 (6.35–7.11)	12.79 (12.74–13.45)	8.99 (8.88–9.1)	11.91 (11.91–12.11)	*, †, ‡
	**IP-10**	238.36 (228.03–242.36)	257.46 (253.73–272.04)	216.56 (210.26–244.94)	145.72 (136.5–157.56)	*, †, ‡
	**MCP-1**	111.40 (98.25–115.35)	249.95 (240.64–303.02)	1399.73 (983.82–1443.44)	2687.61 (2447.81–2832.52)	*, †, ‡
	**MIP-1α**	20.11 (18.01–24.46)	176.13 (172.82–217.53)	248.09 (168.89–283.43)	525.93 (509.38–552.97)	*, †, ‡
	**MIP-1β**	130.16 (115.28–144.06)	752.4 (676.59–814.62)	187.55 (183.67–204.07)	587.40 (538.08–592.08)	*, ‡

**THP-1 Analyte Profile**. Cytokines and chemokines levels were measured in the respective PBS, WTC-PM100μg/mL, LPA500µM, and WTC-PM100μg/mL/LPA exposed THP-1-derived cell culture supernatants. All values represented in pg/mL. Bold values represented medians while values in parentheses represent the first and third quartile range. Minimum Detectable Concentration were used for Lower Limit of Detection. Significance was assessed by Mann-Whitney U test. *: *p* < 0.05 comparing PBS and WTC-PM; †: *p* < 0.05 comparing PBS and LPA; ‡: *p* < 0.05 comparing WTC-PM and WTC-PM/LPA.

**Table 2:** Analyte Profile of RAW265.7 Cells.

	Analyte	PBS	WTC-PM_100μ__g/mL_	LPA	WTC-PM_100μ__g/mL_ + LPA	*p*
**Cytokine**	**G-CSF**	80.20 (43.71–94.81)	2728.19 (2609.32–3177.45)	50.55 (49.12–65.04)	2669.91 (2411.36–2814.15)	*
	**GM-CSF**	7.96 (3.98–10.21)	21.69 (18.83–21.69)	0.00 (0.00–2.4)	12.45 (10.21–15.72)	*
	**IFN-g**	0 (0–0)	4.04 (3.87–4.14)	2.44 (2.30–2.66)	3.83 (3.49–5.37)	*, †
	**IL-1a**	8.07 (4.04–9.30)	40.23 (34.63–43.20)	12.42 (11.48–14.69)	59.04 (58.69–62.51)	*, ‡
	**IL-4**	2.43 (2.41–2.43)	2.57 (2.52–2.58)	2.43 (2.43–2.45)	2.49 (2.46–2.52)	*
	**IL-5**	0 (0–0)	3.95 (2.69–4.11)	0.50 (0.25–1.07)	1.22 (0.61–1.53)	*
	**IL-6**	1.02 (0.51–1.13)	23.02 (21.79–30.13)	0.32 (0.29–0.40)	5.17 (4.87–5.59)	*, ‡
	**IL-7**	3.27 (3.17–3.36)	3.84 (3.51–3.95)	3.10 (3.02–3.19)	3.44 (3.36–3.47)	
	**IL-9**	57.24 (37.37–57.24)	86.70 (84.98–88.38)	99.82 (84.38–123.08)	86.70 (83.26–93.26)	*, †
	**IL-13**	10.88 (10.18–10.88)	15.13 (15.13–17.27)	12.30 (11.59–13.01)	16.55 (16.55–16.55)	*
	**IL-15**	1.00 (0.00–2.08)	17.79 (11.86–20.28)	2.38 (1.46–2.38)	9.36 (4.68–11.06)	*
	**IL-17**	2.13 (2.10–2.17)	3.60 (3.58–3.99)	2.67 (2.56–2.78)	3.03 (2.99–2.03)	*, †, ‡
	**TNF-a**	31.50 (29.96–31.64)	425.42 (410.97–474.19)	292.78 (273.69–302.52)	584.33 (538.91–598.24)	*, †
	**IL-10**	1.85 (1.70–1.85)	7.77 (7.53–8.18)	6.00 (5.20–6.16)	26.78 (25.48–27.27)	*, ‡
	**IL-12(p40)**	10.50 (10.50–10.50)	0.49 (0.25–0.66)	0 (0–0.03)	0 (0–0.14)	
	**IL-12(p70)**	0.40 (0.40–0.40)	0.40 (0.40–2.51)	0.40 (0.40–0.40)	1.40 (0.70–2.06)	
**Chemokine**	**IP-10**	66.24 (65.56–69.22)	90.06 (82.55–110.61)	58.72 (54.7–59.24)	46.83 (40.20–47.04)	*, †, ‡
	**KC**	1.74 (1.66–1.79)	3.92 (3.62–4.11)	1.83 (1.65–1.92)	3.69 (3.58–4.18)	*
	**MCP-1**	886.86 (818.32–913.08)	1824.05 (1764.68–1949.54)	1810.84 (1749.61–1863.66)	2101.59 (2077.03–2203.92)	*, †, ‡
	**RANTES**	4.72 (4.63–4.74)	46.72 (43.50–50.28)	4.50 (4.49–4.64)	10.72 (10.04–13.95)	*, ‡
**Other**	**NF-κB**	0.31 (0.31–0.38)	0.46 (0.40–0.46)	0.37 (0.35–0.42)	0.35 (0.35–0.39)	†

**RAW Analyte Profile.** Analyte levels were measured in the respective PBS, WTC-PM_100μg/mL_, LPA_500µM_, and WTC-PM_100μg/mL_/LPA exposed RAW264.7 cell culture supernatants. All values, excluding NF-κB which is in absorbance, represented in pg/mL. Bold values represented medians while values in parentheses represent the first and third quartile range. Minimum Detectable Concentration were used for Lower Limit of Detection. Significance was assessed by Mann-Whitney U test. *: *p* < 0.05 comparing PBS and WTC-PM; †: *p* < 0.05 comparing PBS and LPA; ‡: *p* < 0.05 comparing WTC-PM and WTC-PM/LPA.

**Table 3:** Analytes available in the metabolomics subcohort.

Analyte (pg/mL)	Controls (*n* = 15)	WTC-LI (*n* = 15)
**GM-CSF**	24.94 (15.16–64.70)	30.05 (22.34–84.34)
**IL-10**	14.02 (3.78–27.69)	10.80 (4.03–32.00)
**IL-8**	12.26 (10.46–36.64)	14.45 (12.88–24.06)
**MCP-1 ^a^**	398.23 (314.11–493.11)	589.41 (495.64–1087.35)
**MIP-1α**	28.11 (17.12–38.38)	26.32 (16.35–36.83)
**IL-1α**	8.62 (3.20–28.57)	4.83 (0.20–36.56)
**TNF-α**	5.83 (4.35–8.84)	7.29 (6.38–9.24)
**LPA**	12.46 (4.73–27.72)	12.02 (8.70–51.94)
**RAGE**	80.59 (68.92–86.70)	77.77 (60.84–100.13)

Values in median (IQR) [9,15,31,67,68,73,79]. ^a^ Significant by Mann–Whitney.

## Abbreviations

AGE	Advanced glycation end-products
Ager	Murine RAGE gene
Ager-/-	RAGE-deficient mice
Akt	Protein kinase B
AP-1	Activator protein 1
BCAA	Branched-chain amino acids
COPD	Chronic obstructive pulmonary disease
EGF	Epidermal growth factor
ELISA	Enzyme-linked immunosorbent assay
ERK	Extracellular-signal-regulated kinase
FCS	Fetal calf serum
FDNY	Fire Department of New York
FEV1	Forced expiratory volume
FEV1%predicted < LLN	Percent of predicted forced expiratory volume less than the lower limit of normal
FGF	fibroblast growth factor
Flt	Fms-like tyrosine kinase
GAPDH	Glyceraldehyde 3-phosphate dehydrogenase
GM-CSF	Granulocyte/macrophage-colony-stimulating factor
GPC	Glycero-phosphatidylcholines
HDL	High-density lipoprotein
IFN	Interferon
IgG	Immunoglobulin G
IL	Interleukin
IP	IFNγ-induced protein
JNK	c-Jun N-terminal kinase
KC	Chemokine kigand 1 (murine)
LDL	Low-density lipoprotein
LPA	Lysophosphatidic acid
MA	Media alone
MCP	Monocyte chemoattractant protein
MDC	Macrophage-derived chemokine
MetSyn	Metabolic syndrome
MIP	Macrophage inflammatory protein
NF-κB	Nuclear factor kappa light-chain-enhancer of activated B cells
OAD	Obstructive airway disease
*p*-Akt	Phosphorylated protein kinase
PBS	Phosphate-buffered saline
PDGF	Platelet-derived growth factor
PLA2	Phospholipase 2
PM	Particulate matter
PMA	Phorbol-12-myristate-13-acetate
PPAR-γ	Peroxisome proliferator-activated receptor gamma
p-STAT	Phosphorylated signal transducer and activator of transcription
RAGE	Receptor for advanced glycation end-products (membrane-bound)
RANTES	Regulated upon activation, normal T cell expressed, and secreted
sCD40L	Soluble cluster of differentiation-40 ligand
sIL-2Rα	Secreted interleukin-2 receptor alpha
SDS-PAGE	Sodium dodecyl sulfate–polyacrylamide gel electrophoresis
SP-1	Specificity protein 1
sRAGE	Soluble RAGE
STAT	Signal transducer and activator of transcription
TGF	Tumor growth factor
TLR	Toll-like receptor
TNF	Tumor necrosis factor
VEGF	Vascular endothelial growth factor
WTC-HP	WTC Health Program
WTC-LI	World Trade Center—lung injury
WTC-PM	World Trade Center—particulate matter

## References

[1] Sint T., Donohue J.F., Ghio A.J. (2008). Ambient air pollution particles and the acute exacerbation of chronic obstructive pulmonary disease. Inhal. Toxicol..

[2] Ling S.H., van Eeden S.F. (2009). Particulate matter air pollution exposure: Role in the development and exacerbation of chronic obstructive pulmonary disease. Int. J. Chronic Obstr. Pulm. Dis..

[3] Dockery D.W., Pope C.A., Xu X., Spengler J.D., Ware J.H., Fay M.E., Ferris B.G., Speizer F.E. (1993). An association between air pollution and mortality in six U.S. cities. N. Engl. J. Med..

[4] Schwartz J. (1995). Short term fluctuations in air pollution and hospital admissions of the elderly for respiratory disease. Thorax.

[5] Brook R.D., Rajagopalan S., Pope C.A., Brook J.R., Bhatnagar A., Diez-Roux A.V., Holguin F., Hong Y., Luepker R.V., Mittleman M.A. (2010). Particulate matter air pollution and cardiovascular disease: An update to the scientific statement from the American Heart Association. Circulation.

[6] Shanley R.P., Hayes R.B., Cromar K.R., Ito K., Gordon T., Ahn J. (2016). Particulate Air Pollution and Clinical Cardiovascular Disease Risk Factors. Epidemiology (Cambridge, Mass.).

[7] Leone N., Courbon D., Thomas F., Bean K., Jego B., Leynaert B., Guize L., Zureik M. (2009). Lung function impairment and metabolic syndrome: The critical role of abdominal obesity. Am. J. Respir. Crit. Care Med..

[8] Kwon S., Crowley G., Caraher E.J., Haider S.H., Lam R., Veerappan A., Yang L., Liu M., Zeig-Owens R., Schwartz T.M. (2019). Validation of Predictive Metabolic Syndrome Biomarkers of World Trade Center Lung Injury: A 16-Year Longitudinal Study. Chest.

[9] Naveed B., Weiden M.D., Kwon S., Gracely E.J., Comfort A.L., Ferrier N., Kasturiarachchi K.J., Cohen H.W., Aldrich T.K., Rom W.N. (2012). Metabolic syndrome biomarkers predict lung function impairment: A nested case-control study. Am. J. Respir. Crit. Care Med..

[10] Kwon S., Echevarria G.C., Cho S., Tsukiji J., Rom W.N., Prezant D.J., Schmidt A., Weiden M.D., Nolan A. (2014). Soluble Rage, Mmp-9 And Crp Are Predictive Of Particulate Matter Induced Lung Disease In Wtc Exposed Firefighters. Am. J. Resp. Crit. Care Med..

[11] Weiden M.D., Kwon S., Caraher E., Berger K.I., Reibman J., Rom W.N., Prezant D.J., Nolan A. (2015). Biomarkers of World Trade Center Particulate Matter Exposure: Physiology of Distal Airway and Blood Biomarkers that Predict FEV(1) Decline. Semin. Respir. Crit. Care Med..

[12] Holguin F. (2012). The metabolic syndrome as a risk factor for lung function decline. Am. J. Respir. Crit. Care Med..

[13] Balmes J.R. (2012). Can we predict who will develop chronic sequelae of acute inhalational injury?. Chest.

[14] Antao V.C. (2013). The World Trade Center disaster: A tragic source of medical advancement. Eur. Respir. J..

[15] Caraher E.J., Kwon S., Haider S.H., Crowley G., Lee A., Ebrahim M., Zhang L., Chen L.C., Gordon T., Liu M. (2017). Receptor for advanced glycation end-products and World Trade Center particulate induced lung function loss: A case-cohort study and murine model of acute particulate exposure. PLoS ONE.

[16] Zhao Y., Natarajan V. (2013). Lysophosphatidic acid (LPA) and its receptors: Role in airway inflammation and remodeling. Biochim. Biophys. Acta.

[17] Haider S.H., Veerappan A., Crowley G., Ostrofsky D., Mikhail M., Lam R., Wang Y., Sunseri M., Kwon S., Prezant D.J. (2020). MultiOMICs of WTC-Particulate Induced Persistent Airway Hyperreactivity: Role of Receptor for Advanced Glycation End Products. Am. J. Respir. Cell Mol. Biol..

[18] Yan S.F., Ramasamy R., Schmidt A.M. (2010). The RAGE axis: A fundamental mechanism signaling danger to the vulnerable vasculature. Circ. Res..

[19] Koyama H., Yamamoto H., Nishizawa Y. (2007). Endogenous Secretory RAGE as a Novel Biomarker for Metabolic Syndrome and Cardiovascular Diseases. Biomark. Insights.

[20] Hancock D.B., Eijgelsheim M., Wilk J.B., Gharib S.A., Loehr L.R., Marciante K.D., Franceschini N., van Durme Y.M., Chen T.H., Barr R.G. (2010). Meta-analyses of genome-wide association studies identify multiple loci associated with pulmonary function. Nat. Genet..

[21] Repapi E., Sayers I., Wain L.V., Burton P.R., Johnson T., Obeidat M., Zhao J.H., Ramasamy A., Zhai G., Vitart V. (2010). Genome-wide association study identifies five loci associated with lung function. Nat. Genet..

[22] Beucher J., Boelle P.Y., Busson P.F., Muselet-Charlier C., Clement A., Corvol H., French C.F.M.G.S.I. (2012). AGER -429T/C is associated with an increased lung disease severity in cystic fibrosis. PLoS ONE.

[23] Miller S., Henry A.P., Hodge E., Kheirallah A.K., Billington C.K., Rimington T.L., Bhaker S.K., Obeidat M., Melen E., Merid S.K. (2016). The Ser82 RAGE Variant Affects Lung Function and Serum RAGE in Smokers and sRAGE Production In Vitro. PLoS ONE.

[24] Huang J.S., Guh J.Y., Chen H.C., Hung W.C., Lai Y.H., Chuang L.Y. (2001). Role of receptor for advanced glycation end-product (RAGE) and the JAK/STAT-signaling pathway in AGE-induced collagen production in NRK-49F cells. J. Cell Biochem..

[25] Oczypok E.A., Perkins T.N., Oury T.D. (2017). All the “RAGE” in lung disease: The receptor for advanced glycation endproducts (RAGE) is a major mediator of pulmonary inflammatory responses. Paediatr. Respir. Rev..

[26] Tobon-Velasco J.C., Cuevas E., Torres-Ramos M.A. (2014). Receptor for AGEs (RAGE) as mediator of NF-kB pathway activation in neuroinflammation and oxidative stress. CNS Neurol. Disord. Drug Targets.

[27] Wu L., Ma L., Nicholson L.F., Black P.N. (2011). Advanced glycation end products and its receptor (RAGE) are increased in patients with COPD. Respir. Med..

[28] Polverino F., Celli B.R., Owen C.A. (2018). COPD as an endothelial disorder: Endothelial injury linking lesions in the lungs and other organs? (2017 Grover Conference Series). Pulm. Circ..

[29] Sukkar M.B., Ullah M.A., Gan W.J., Wark P.A., Chung K.F., Hughes J.M., Armour C.L., Phipps S. (2012). RAGE: A new frontier in chronic airways disease. Br. J. Pharmacol..

[30] Sukkar M.B., Wood L.G., Tooze M., Simpson J.L., McDonald V.M., Gibson P.G., Wark P.A. (2012). Soluble RAGE is deficient in neutrophilic asthma and COPD. Eur. Respir. J..

[31] Tsukiji J., Cho S.J., Echevarria G.C., Kwon S., Joseph P., Schenck E.J., Naveed B., Prezant D.J., Rom W.N., Schmidt A.M. (2014). Lysophosphatidic acid and apolipoprotein A1 predict increased risk of developing World Trade Center-lung injury: A nested case-control study. Biomarkers.

[32] Moolenaar W.H., van Meeteren L.A., Giepmans B.N. (2004). The ins and outs of lysophosphatidic acid signaling. BioEssays News Rev. Mol. Cell. Dev. Biol..

[33] Lin M.E., Herr D.R., Chun J. (2010). Lysophosphatidic acid (LPA) receptors: Signaling properties and disease relevance. Prostaglandins Other Lipid Mediat..

[34] Murph M., Mills G.B. (2007). Targeting the lipids LPA and S1P and their signalling pathways to inhibit tumour progression. Expert Rev. Mol. Med..

[35] Smyth S.S., Cheng H.Y., Miriyala S., Panchatcharam M., Morris A.J. (2008). Roles of lysophosphatidic acid in cardiovascular physiology and disease. Biochim. Biophys. Acta.

[36] Chuyen N.V. (2006). Toxicity of the AGEs generated from the Maillard reaction: On the relationship of food-AGEs and biological-AGEs. Mol. Nutr. Food Res..

[37] Cerami C., Founds H., Nicholl I., Mitsuhashi T., Giordano D., Vanpatten S., Lee A., AlAbed Y., Vlassara H., Bucala R. (1997). Tobacco smoke is a source of toxic reactive glycation products. Proc. Natl. Acad. Sci. USA.

[38] Aoki J., Inoue A., Okudaira S. (2008). Two pathways for lysophosphatidic acid production. Biochim. Biophys. Acta.

[39] Mills G.B., Moolenaar W.H. (2003). The emerging role of lysophosphatidic acid in cancer. Nat. Rev. Cancer.

[40] Georas S.N. (2009). Lysophosphatidic acid and autotaxin: Emerging roles in innate and adaptive immunity. Immunol. Res..

[41] Pamuklar Z., Federico L., Liu S., Umezu-Goto M., Dong A., Panchatcharam M., Fulkerson Z., Berdyshev E., Natarajan V., Fang X. (2009). Autotaxin/lysopholipase D and lysophosphatidic acid regulate murine hemostasis and thrombosis. J. Biol. Chem..

[42] Brindley D.N., Pilquil C. (2009). Lipid phosphate phosphatases and signaling. J. Lipid Res..

[43] Rodriguez-Roisin R., Drakulovic M., Rodriguez D.A., Roca J., Barbera J.A., Wagner P.D. (2009). Ventilation-perfusion imbalance and chronic obstructive pulmonary disease staging severity. J. Appl. Physiol..

[44] Liebow A.A. (1959). Pulmonary emphysema with special reference to vascular changes. Am. Rev. Respir. Dis..

[45] Caplan-Shaw C.E., Yee H., Rogers L., Abraham J.L., Parsia S.S., Naidich D.P., Borczuk A., Moreira A., Shiau M.C., Ko J.P. (2011). Lung pathologic findings in a local residential and working community exposed to World Trade Center dust, gas, and fumes. J. Occup. Environ. Med..

[46] King M.S., Eisenberg R., Newman J.H., Tolle J.J., Harrell F.E., Nian H., Ninan M., Lambright E.S., Sheller J.R., Johnson J.E. (2011). Constrictive bronchiolitis in soldiers returning from Iraq and Afghanistan. N. Engl. J. Med..

[47] Noguchi K., Herr D., Mutoh T., Chun J. (2009). Lysophosphatidic acid (LPA) and its receptors. Curr. Opin. Pharmacol..

[48] Choi J.W., Herr D.R., Noguchi K., Yung Y.C., Lee C.W., Mutoh T., Lin M.E., Teo S.T., Park K.E., Mosley A.N. (2010). LPA receptors: Subtypes and biological actions. Annu. Rev. Pharmacol. Toxicol..

[49] Chun J., Hla T., Lynch K.R., Spiegel S., Moolenaar W.H. (2010). International Union of Basic and Clinical Pharmacology. LXXVIII. Lysophospholipid receptor nomenclature. Pharmacol. Rev..

[50] McIntyre T.M., Pontsler A.V., Silva A.R., St Hilaire A., Xu Y., Hinshaw J.C., Zimmerman G.A., Hama K., Aoki J., Arai H. (2003). Identification of an intracellular receptor for lysophosphatidic acid (LPA): LPA is a transcellular PPARgamma agonist. Proc. Natl. Acad. Sci. USA.

[51] Stapleton C.M., Mashek D.G., Wang S., Nagle C.A., Cline G.W., Thuillier P., Leesnitzer L.M., Li L.O., Stimmel J.B., Shulman G.I. (2011). Lysophosphatidic acid activates peroxisome proliferator activated receptor-gamma in CHO cells that over-express glycerol 3-phosphate acyltransferase-1. PLoS ONE.

[52] Bodine B.G., Bennion B.G., Leatham E., Jimenez F.R., Wright A.J., Jergensen Z.R., Erickson C.J., Jones C.M., Johnson J.P., Knapp S.M. (2014). Conditionally induced RAGE expression by proximal airway epithelial cells in transgenic mice causes lung inflammation. Respir. Res..

[53] Wolf L., Herr C., Niederstrasser J., Beisswenger C., Bals R. (2017). Receptor for advanced glycation endproducts (RAGE) maintains pulmonary structure and regulates the response to cigarette smoke. PLoS ONE.

[54] Weiden M.D., Naveed B., Kwon S., Segal L.N., Cho S.J., Tsukiji J., Kulkarni R., Comfort A.L., Kasturiarachchi K.J., Prophete C. (2012). Comparison of WTC dust size on macrophage inflammatory cytokine release in vivo and in vitro. PLoS ONE.

[55] Becker S., Dailey L.A., Soukup J.M., Grambow S.C., Devlin R.B., Huang Y.C. (2005). Seasonal variations in air pollution particle-induced inflammatory mediator release and oxidative stress. Environ. Health Perspect..

[56] Theus S.A., Cave M.D., Eisenach K.D. (2004). Activated THP-1 cells: An attractive model for the assessment of intracellular growth rates of Mycobacterium tuberculosis isolates. Infect. Immun..

[57] Smith L.S., Gharib S.A., Frevert C.W., Martin T.R. (2010). Effects of Age on the Synergistic Interactions between Lipopolysaccharide and Mechanical Ventilation in Mice. Am. J. Resp. Cell Mol..

[58] McGee J.K., Chen L.C., Cohen M.D., Chee G.R., Prophete C.M., Haykal-Coates N., Wasson S.J., Conner T.L., Costa D.L., Gavett S.H. (2003). Chemical analysis of World Trade Center fine particulate matter for use in toxicologic assessment. Environ. Health Perspect..

[59] Michalczyk A., Budkowska M., Dolegowska B., Chlubek D., Safranow K. (2017). Lysophosphatidic acid plasma concentrations in healthy subjects: Circadian rhythm and associations with demographic, anthropometric and biochemical parameters. Lipids Health Dis..

[60] Mathew D., Kremer K.N., Strauch P., Tigyi G., Pelanda R., Torres R.M. (2019). LPA5 Is an Inhibitory Receptor That Suppresses CD8 T-Cell Cytotoxic Function via Disruption of Early TCR Signaling. Front. Immunol..

[61] Gavett S.H., Haykal-Coates N., Highfill J.W., Ledbetter A.D., Chen L.C., Cohen M.D., Harkema J.R., Wagner J.G., Costa D.L. (2003). World Trade Center fine particulate matter causes respiratory tract hyperresponsiveness in mice. Environ. Health Perspect..

[62] Gold J.A., Parsey M., Hoshino Y., Hoshino S., Nolan A., Yee H., Tse D.B., Weiden M.D. (2003). CD40 contributes to lethality in acute sepsis: In vivo role for CD40 in innate immunity. Infect. Immun..

[63] Kobayashi H., Nolan A., Naveed B., Hoshino Y., Segal L.N., Fujita Y., Rom W.N., Weiden M.D. (2011). Neutrophils activate alveolar macrophages by producing caspase-6-mediated cleavage of IL-1 receptor-associated kinase-M. J. Immunol..

[64] Nolan A., Weiden M., Kelly A., Hoshino Y., Hoshino S., Mehta N., Gold J.A. (2008). CD40 and CD80/86 act synergistically to regulate inflammation and mortality in polymicrobial sepsis. Am. J. Respir. Crit. Care Med..

[65] Nolan A., Weiden M.D., Hoshino Y., Gold J.A. (2004). Cd40 but not CD154 knockout mice have reduced inflammatory response in polymicrobial sepsis: A potential role for Escherichia coli heat shock protein 70 in CD40-mediated inflammation in vivo. Shock.

[66] Rasband W.S. (1997–2018). ImageJ.

[67] Nolan A., Naveed B., Comfort A.L., Ferrier N., Hall C.B., Kwon S., Kasturiarachchi K.J., Cohen H.W., Zeig-Owens R., Glaser M.S. (2012). Inflammatory biomarkers predict airflow obstruction after exposure to World Trade Center dust. Chest.

[68] Weiden M.D., Naveed B., Kwon S., Cho S.J., Comfort A.L., Prezant D.J., Rom W.N., Nolan A. (2013). Cardiovascular biomarkers predict susceptibility to lung injury in World Trade Center dust-exposed firefighters. Eur. Respir. J..

[69] Cho S., Echevarria G.C., Kwon S., Naveed B., Schenck E., Tsukiji J., Rom W.N., Prezant D.J., Nolan A. (2018). One Airway: Biomarkers Of Protection From Upper And Lower Airway Injury After World Trade Center Exposure. Respir. Med..

[70] Banauch G.I., Dhala A., Prezant D.J. (2005). Pulmonary disease in rescue workers at the World Trade Center site. Curr. Opin. Pulm. Med..

[71] Prezant D.J., Weiden M., Banauch G.I., McGuinness G., Rom W.N., Aldrich T.K., Kelly K.J. (2002). Cough and bronchial responsiveness in firefighters at the World Trade Center site. N. Engl. J. Med..

[72] Weiden M.D., Ferrier N., Nolan A., Rom W.N., Comfort A., Gustave J., Zeig-Owens R., Zheng S., Goldring R.M., Berger K.I. (2010). Obstructive airways disease with air trapping among firefighters exposed to World Trade Center dust. Chest.

[73] Crowley G., Kwon S., Haider S.H., Caraher E.J., Lam R., St-Jules D.E., Liu M., Prezant D.J., Nolan A. (2018). Metabolomics of World Trade Center-Lung Injury: A machine learning approach. BMJ Open Respir. Res..

[74] Crowley G., Kwon S., Ostrofsky D.F., Clementi E.A., Haider S.H., Caraher E.J., Lam R., St-Jules D.E., Liu M., Prezant D.J. (2019). Assessing the Protective Metabolome Using Machine Learning in World Trade Center Particulate Exposed Firefighters at Risk for Lung Injury. Sci. Rep..

[75] Kwon S., Crowley G., Mikhail M., Lam R., Clementi E., Zeig-Owens R., Schwartz T.M., Liu M., Prezant D.J., Nolan A. (2019). Metabolic Syndrome Biomarkers of World Trade Center Airway Hyperreactivity: A 16-Year Prospective Cohort Study. Int. J. Environ. Res. Public Health.

[76] Farzi A., Reichmann F., Meinitzer A., Mayerhofer R., Jain P., Hassan A.M., Frohlich E.E., Wagner K., Painsipp E., Rinner B. (2014). Synergistic effects of NOD1 or NOD2 and TLR4 activation on mouse sickness behavior in relation to immune and brain activity markers. Brain Behav. Immun..

[77] Gunja N.J., Athanasiou K.A. (2010). Additive and synergistic effects of bFGF and hypoxia on leporine meniscus cell-seeded PLLA scaffolds. J. Tissue Eng. Regen. Med..

[78] Haider S.H., Kwon S., Lam R., Lee A.K., Caraher E.J., Crowley G., Zhang L., Schwartz T.M., Zeig-Owens R., Liu M. (2018). Predictive Biomarkers of Gastroesophageal Reflux Disease and Barrett’s Esophagus in World Trade Center Exposed Firefighters: A 15 Year Longitudinal Study. Sci. Rep..

[79] Zhang L., Haider S.H., Crowley G., Lam R., Kwon S., Chen L.C., Schmidt A.M., Prezant D.J., Nolan A. (2017). World Trade Center Particulates And Lysophosphatdic Acid: Co-Exposure Induces Inflammatory Mediators. Am. J. Respir. Crit. Care Med..

[80] Caraher E.J., Kwon S., Lee A.K., Chen L.-C., Gordon T., Prezant D.J., Rom W.N., Weiden M.D., Nolan A. (2015). Additive and Synergistic Effects of LPA in World Trade Center Particulate Matter-Induced Inflammation. Am. J. Respir. Crit. Care Med..

[81] Crowley G., Kwon S., Haider S., Caraher E.J., Lam R., Liu M., Prezant D.J., Nolan A. (2018). Metabolite and Biomarker Predictors of World Trade Center-Lung Injury: An Integrated Multiplatform Machine Learning Approach. Am. J. Resp. Crit. Care.

[82] Huang W., Wang L., Li J.P., Liu M.C., Xu H.B., Liu S.C., Chen J., Zhang Y., Morishita M., Bard R.L. (2018). Short-Term Blood Pressure Responses to Ambient Fine Particulate Matter Exposures at the Extremes of Global Air Pollution Concentrations. Am. J. Hypertens..

[83] Eze I.C., Schaffner E., Foraster M., Imboden M., von Eckardstein A., Gerbase M.W., Rothe T., Rochat T., Kunzli N., Schindler C. (2015). Long-Term Exposure to Ambient Air Pollution and Metabolic Syndrome in Adults. PLoS ONE.

[84] Brook R.D., Cakmak S., Turner M.C., Brook J.R., Crouse D.L., Peters P.A., van Donkelaar A., Villeneuve P.J., Brion O., Jerrett M. (2013). Long-term fine particulate matter exposure and mortality from diabetes in Canada. Diabetes Care.

[85] Traves S.L., Culpitt S.V., Russell R.E., Barnes P.J., Donnelly L.E. (2002). Increased levels of the chemokines GROalpha and MCP-1 in sputum samples from patients with COPD. Thorax.

[86] Di Stefano A., Coccini T., Roda E., Signorini C., Balbi B., Brunetti G., Ceriana P. (2018). Blood MCP-1 levels are increased in chronic obstructive pulmonary disease patients with prevalent emphysema. Int. J. Chron. Obstruct. Pulmon. Dis..

[87] Basta G. (2008). Receptor for advanced glycation endproducts and atherosclerosis: From basic mechanisms to clinical implications. Atherosclerosis.

[88] Fritz G. (2011). RAGE: A single receptor fits multiple ligands. Trends Biochem. Sci..

[89] Simeonova P.P., Luster M.I. (1995). Iron and reactive oxygen species in the asbestos-induced tumor necrosis factor-alpha response from alveolar macrophages. Am. J. Respir. Cell Mol. Biol..

[90] Funahashi S., Okazaki Y., Ito D., Asakawa A., Nagai H., Tajima M., Toyokuni S. (2015). Asbestos and multi-walled carbon nanotubes generate distinct oxidative responses in inflammatory cells. J. Clin. Biochem. Nutr..

[91] Hart G.A., Hesterberg T.W. (1998). In vitro toxicity of respirable-size particles of diatomaceous earth and crystalline silica compared with asbestos and titanium dioxide. J. Occup. Environ. Med..

[92] Huang S.X., Jaurand M.C., Kamp D.W., Whysner J., Hei T.K. (2011). Role of mutagenicity in asbestos fiber-induced carcinogenicity and other diseases. J. Toxicol. Environ. Health B Crit. Rev..

[93] Hesterberg T.W., Chase G., Axten C., Miller W.C., Musselman R.P., Kamstrup O., Hadley J., Morscheidt C., Bernstein D.M., Thevenaz P. (1998). Biopersistence of synthetic vitreous fibers and amosite asbestos in the rat lung following inhalation. Toxicol. Appl. Pharmacol..

[94] Jaishankar M., Tseten T., Anbalagan N., Mathew B.B., Beeregowda K.N. (2014). Toxicity, mechanism and health effects of some heavy metals. Interdiscip. Toxicol..

[95] Simeonova P.P., Toriumi W., Kommineni C., Erkan M., Munson A.E., Rom W.N., Luster M.I. (1997). Molecular regulation of IL-6 activation by asbestos in lung epithelial cells: Role of reactive oxygen species. J. Immunol..

[96] Satpathy S.R., Jala V.R., Bodduluri S.R., Krishnan E., Hegde B., Hoyle G.W., Fraig M., Luster A.D., Haribabu B. (2015). Crystalline silica-induced leukotriene B4-dependent inflammation promotes lung tumour growth. Nat. Commun..

[97] Stone V., Brown D.M., Watt N., Wilson M., Donaldson K., Ritchie H., MacNee W. (2000). Ultrafine Particle-Mediated Activation of Macrophages: Intracellular Calcium Signaling and Oxidative Stress. Inhal. Toxicol..

[98] Boulanger G., Andujar P., Pairon J.C., Billon-Galland M.A., Dion C., Dumortier P., Brochard P., Sobaszek A., Bartsch P., Paris C. (2014). Quantification of short and long asbestos fibers to assess asbestos exposure: A review of fiber size toxicity. Environ. Health.

[99] Msiska Z., Pacurari M., Mishra A., Leonard S.S., Castranova V., Vallyathan V. (2010). DNA double-strand breaks by asbestos, silica, and titanium dioxide: Possible biomarker of carcinogenic potential?. Am. J. Respir. Cell Mol. Biol..

[100] Chen Q., Marsh J., Ames B., Mossman B. (1996). Detection of 8-oxo-2’-deoxyguanosine, a marker of oxidative DNA damage, in culture medium from human mesothelial cells exposed to crocidolite asbestos. Carcinogenesis.

[101] An D., Hao F., Zhang F.Q., Kong W., Chun J., Xu X.M., Cui M.Z. (2017). CD14 is a key mediator of both lysophosphatidic acid and lipopolysaccharide induction of foam cell formation. J. Biol. Chem..

[102] Chen L., Zhang J., Deng X., Liu Y., Yang X., Wu Q., Yu C. (2017). Lysophosphatidic acid directly induces macrophage-derived foam cell formation by blocking the expression of SRBI. Biochem. Biophys. Res. Commun..

[103] Ray R., Rai V. (2017). Lysophosphatidic acid converts monocytes into macrophages in both mice and humans. Blood.

[104] Bekkering S., Quintin J., Joosten L.A., van der Meer J.W., Netea M.G., Riksen N.P. (2014). Oxidized low-density lipoprotein induces long-term proinflammatory cytokine production and foam cell formation via epigenetic reprogramming of monocytes. Arterioscler. Thromb. Vasc. Biol..

[105] Bradford E., Jacobson S., Varasteh J., Comellas A.P., Woodruff P., O’Neal W., DeMeo D.L., Li X., Kim V., Cho M. (2017). The value of blood cytokines and chemokines in assessing COPD. Respir. Res..

[106] Ulrey C.L., Liu L., Andrews L.G., Tollefsbol T.O. (2005). The impact of metabolism on DNA methylation. Hum. Mol. Genet.

[107] Zaina S., Lindholm M.W., Lund G. (2005). Nutrition and aberrant DNA methylation patterns in atherosclerosis: More than just hyperhomocysteinemia?. J. Nutr..

[108] Lund G., Andersson L., Lauria M., Lindholm M., Fraga M.F., Villar-Garea A., Ballestar E., Esteller M., Zaina S. (2004). DNA methylation polymorphisms precede any histological sign of atherosclerosis in mice lacking apolipoprotein E. J. Biol. Chem..

[109] Castro R., Rivera I., Struys E.A., Jansen E.E., Ravasco P., Camilo M.E., Blom H.J., Jakobs C., Tavares de Almeida I. (2003). Increased homocysteine and S-adenosylhomocysteine concentrations and DNA hypomethylation in vascular disease. Clin. Chem..

[110] Birukova A.A., Starosta V., Tian X., Higginbotham K., Koroniak L., Berliner J.A., Birukov K.G. (2013). Fragmented oxidation products define barrier disruptive endothelial cell response to OxPAPC. Transl. Res..

[111] Hurley B.P., McCormick B.A. (2008). Multiple roles of phospholipase A2 during lung infection and inflammation. Infect. Immun..

[112] Wu T., Ikezono T., Angus C.W., Shelhamer J.H. (1996). Tumor necrosis factor-alpha induces the 85-kDa cytosolic phospholipase A2 gene expression in human bronchial epithelial cells. Biochim. Biophys. Acta.

[113] Bhowmick R., Clark S., Bonventre J.V., Leong J.M., McCormick B.A. (2017). Cytosolic Phospholipase A2alpha Promotes Pulmonary Inflammation and Systemic Disease during Streptococcus pneumoniae Infection. Infect. Immun..

[114] Jemel I., Ii H., Oslund R.C., Payre C., Dabert-Gay A.S., Douguet D., Chargui K., Scarzello S., Gelb M.H., Lambeau G. (2011). Group X secreted phospholipase A2 proenzyme is matured by a furin-like proprotein convertase and releases arachidonic acid inside of human HEK293 cells. J. Biol. Chem..

[115] Pawliczak R., Huang X.L., Nanavaty U.B., Lawrence M., Madara P., Shelhamer J.H. (2002). Oxidative stress induces arachidonate release from human lung cells through the epithelial growth factor receptor pathway. Am. J. Respir. Cell Mol. Biol..

[116] Liu G., Cheresh P., Kamp D.W. (2013). Molecular basis of asbestos-induced lung disease. Annu. Rev. Pathol..

[117] Yan W., Jenkins C.M., Han X., Mancuso D.J., Sims H.F., Yang K., Gross R.W. (2005). The highly selective production of 2-arachidonoyl lysophosphatidylcholine catalyzed by purified calcium-independent phospholipase A2gamma: Identification of a novel enzymatic mediator for the generation of a key branch point intermediate in eicosanoid signaling. J. Biol. Chem..

[118] Rajdl D., Racek J., Trefil L., Stehlik P., Dobra J., Babuska V. (2016). Effect of Folic Acid, Betaine, Vitamin B(6), and Vitamin B12 on Homocysteine and Dimethylglycine Levels in Middle-Aged Men Drinking White Wine. Nutrients.

[119] Lioy P.J., Weisel C.P., Millette J.R., Eisenreich S., Vallero D., Offenberg J., Buckley B., Turpin B., Zhong M., Cohen M.D. (2002). Characterization of the dust/smoke aerosol that settled east of the World Trade Center (WTC) in lower Manhattan after the collapse of the WTC 11 September 2001. Environ. Health Perspect..

[120] World Health Organization 9 out of 10 People Worldwide Breathe Polluted Air, but More Countries are Taking Action. https://www.who.int/news-room/detail/02-05-2018-9-out-of-10-people-worldwide-breathe-polluted-air-but-more-countries-are-taking-action.

[121] Peters U., Suratt B.T., Bates J.H.T., Dixon A.E. (2018). Beyond BMI: Obesity and Lung Disease. Chest.

[122] Saklayen M.G. (2018). The Global Epidemic of the Metabolic Syndrome. Curr. Hypertens. Rep..

[123] Guarnieri M., Balmes J.R. (2014). Outdoor air pollution and asthma. Lancet.

[124] Dixon A.E., Holguin F. (2019). Diet and Metabolism in the Evolution of Asthma and Obesity. Clin. Chest Med..

